# The impact of clinical versus pathological staging in oral cavity carcinoma–a multi-institutional analysis of survival

**DOI:** 10.1186/1916-0216-42-28

**Published:** 2013-04-11

**Authors:** Vincent L Biron, Daniel A O’Connell, Hadi Seikaly

**Affiliations:** 1 Department of Surgery, Division of Otolaryngology-Head and Neck Surgery, University of Alberta, University of Alberta Hospital, Edmonton, AB, 1E4.33 WMC, , Canada

## Abstract

**Objectives:**

To evaluate any disparity in clinical versus pathological TNM staging in oral cavity squamous cell carcinoma (OCSCC) patients and any impact of this on survival.

**Design:**

Demographic, survival, staging, and pathologic data on all patients undergoing surgical treatment for OCSCC in Alberta between 1998 and 2006 was collected. Clinical and pathological TNM staging data were compared. Patients were stratified as pathologically downstaged, upstaged or unchanged.

**Setting:**

Tertiary care centers in Alberta, Canada.

**Main outcome measures:**

Survival differences between groups were analyzed using Kaplan-Meier and Cox regression models.

**Results:**

Patients with clinically early stage tumors were pathologically upstaged in 21.9% of cases and unchanged in 78.1% of cases. Patients with clinically advanced stage tumors were pathologically downstaged in 7.9% of cases and unchanged in 92.1% of cases. Univariate and multivariate estimates of disease-specific survival showed no statistically significant differences in survival when patients were either upstaged or downstaged.

**Conclusions:**

Some disparity exists in clinical versus pathological staging in OCSCC, however, this does not have any significant impact on disease specific survival.

## Introduction

Accurate clinical staging is important for patient counselling, treatment planning, prognostication and the rational design of clinical trials [1]. At the time of diagnosis, treatment strategies are largely based upon clinical staging. In head and neck squamous cell carcinoma, discrepancy between clinical and pathological staging has been reported. Upstaging from early stage N0 neck to node positive neck has been shown to occur in 34–44% of cases and has been shown to have a negative impact on survival [2,3]. This discrepancy is largely attributed to clinical inaccuracy of lymph node staging. Clinical assessment by palpation has been shown to be 60–70% accurate but the incorporation of computed tomography (CT) scanning can improve the accuracy to approximately 90% [2-4].

To our knowledge, the rate at which overall staging discrepancy occurs in oral cavity cancers has not been addressed. We therefore underwent a retrospective, multi-institutional cohort study to investigate the rate of staging discrepancy in OCSCC patients and whether this has any impact on disease specific survival.

## Materials and methods

### Patients

With ethical approval from the Alberta Cancer Board, we obtained an information database containing 560 patients with oral cavity carcinoma diagnosed and treated in Alberta between 1998 and 2006. This database was refined to include solely patients with OCSCC where surgery was included in their treatment pathway (either primary surgery or salvage) such that pathological staging information could be obtained. The database was cross-referenced to patient charts or electronic medical records to verify the integrity of the data, particularly for information involving staging, treatment and dates last known alive. This enabled us to obtain demographic (Table 1), survival, clinical and pathologic staging data on 379 patients. Of these 379 patients, 201 (53.1%) were treated surgically, 171 (45.1%) were treated with surgery and radiotherapy, and 7 (1.8%) were treated with surgery in addition to radiotherapy and surgery. Patients were diagnosed and treated by several Otolaryngology-Head and Neck Surgeons, Radiation Oncologists and Medical Oncologists in tertiary care centres located in Edmonton and Calgary, Alberta, Canada.

**Table 1:** Demographics of 379 patients with oral cavity squamous cell carcinoma

**Age**	
Mean	60.3
Range	26–95
**Gender**	
Male	230 (60.6)
Female	149 (39.4)
**Site**	
Border tongue	63
Tongue NOS	60
FOM NOS	48
Anterior FOM	31
Check mucosa	30
Retromolar area	29
Lower gun	25
Ventral surface tongue	25
Anterior 2/3 Tongue	22
Lateral FOM	14
Mounth NOS	10
Upper Gum	7
Hard Palate	6
Overlaping lesion lip/oral cavity/pharynx	3
Dorsal surface tongue	3
Gum NOS	2
Vestibule of mouth	1

**Figure 1:**
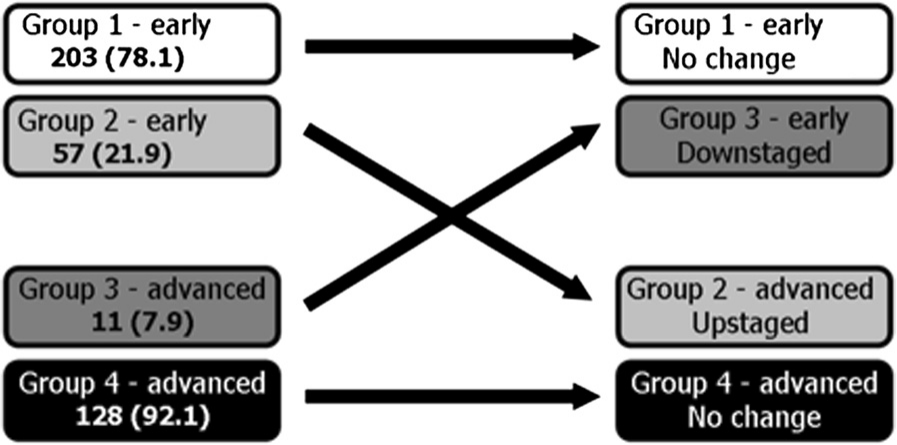
Upstaging and downstaging in early vs late stage oral cavity squamous cell carcinoma in 379 patients.

Overall clinical and pathological TNM staging was compared and tabulated to determine upstaging, downstaging or cases where no stage discrepancy occurred (Table 2). We classified patients into four groups for survival analysis: 1) early stage patients with no pathological stage change, 2) early stage patients upstaged to advanced stage, 3) advanced stage patients with no stage change and 4) advanced stage patients downstaged to early stage (Figure 1).

**Table 2:** Correlation between clinical and pathological tumor staging in 379 patients with oral cavity squamous cell carcinoma

						**Stage discrepancy within clinical stage strata**		
	**p1**	**p2**	**p3**	**p4**	**Totals**	**Upstaged (all)**	**No changed (all)**	**Downstaged (all)**
c1	**109(82.6)**	11(8.3)	4(3.0)	8(6.0)	132	23(17.4)	**109 (82.6)**	-
c2	16(14.0)	**55(48.2)**	20(17.5)	23(20.2)	114	43(37.8)	**55(48.2)**	16(14.0)
c3	4(7.0)	4(7.0)	**25(43.9)**	24(42.1)	57	24(42.1)	**25(43.9)**	8(14.0)
c4	1(1.3)	2(2.6)	4(5.3)	**69(90.8)**	76		**69(90.8)**	7(9.2)

### Survival analysis

All survival analyses were performed using SPSS for Windows version 15 (Chicago, Il). Disease free survival curves were generated using the Kaplan-Meier algorithm as recently described [5]. To determine whether significant differences (p-value < 0.05) were present between these survival curves, we employed the log-rank test. Time zero was defined as the date of diagnosis and surviving patients were included up to the date last known alive, according to time last seen in Otolaryngology-Head and Neck Surgery Clinic from electronic medical records or paper charts. The date and cause of death was obtained as recorded by the Alberta Cancer Board. Multivariate analysis was performed using Cox regression, incorporating patient age and gender as variables.

## Results

Of 379 patients with OCSCC analyzed, 60.6% were male, 39.4% were female and the mean age at diagnosis was 60.3 (Table 1). These patients had tumours in various oral cavity subsites, of which tongue and floor of mouth were most common.

For each patient with an assigned clinical stage, the corresponding pathological stage is summarized in Table 2. Highest congruence between clinical and pathological staging was seen for clinical stages 1 and 4 at 82.6% and 90.8% respectively. Lower levels of correlation were seen for clinical stages 2 (48.2%) and 3 (43.9%). This level of disparity is largely attributed to upstaging, shown in 37.8% of clinically stage 2 patients and 42.1% of stage 3 patients.

Staging discrepancy between early stage (stages 1 and 2) and advanced stage disease (stages 3 and 4) is summarized in Figure 1. Of the clinically early stage patients, 78.1% remained early stage and 21.9% were upstaged to advanced stage following pathological analysis. Of the clinically advanced stage patients, 92.1% remained advanced stage and 7.9% were pathologically downstaged.

**Figure 2:**
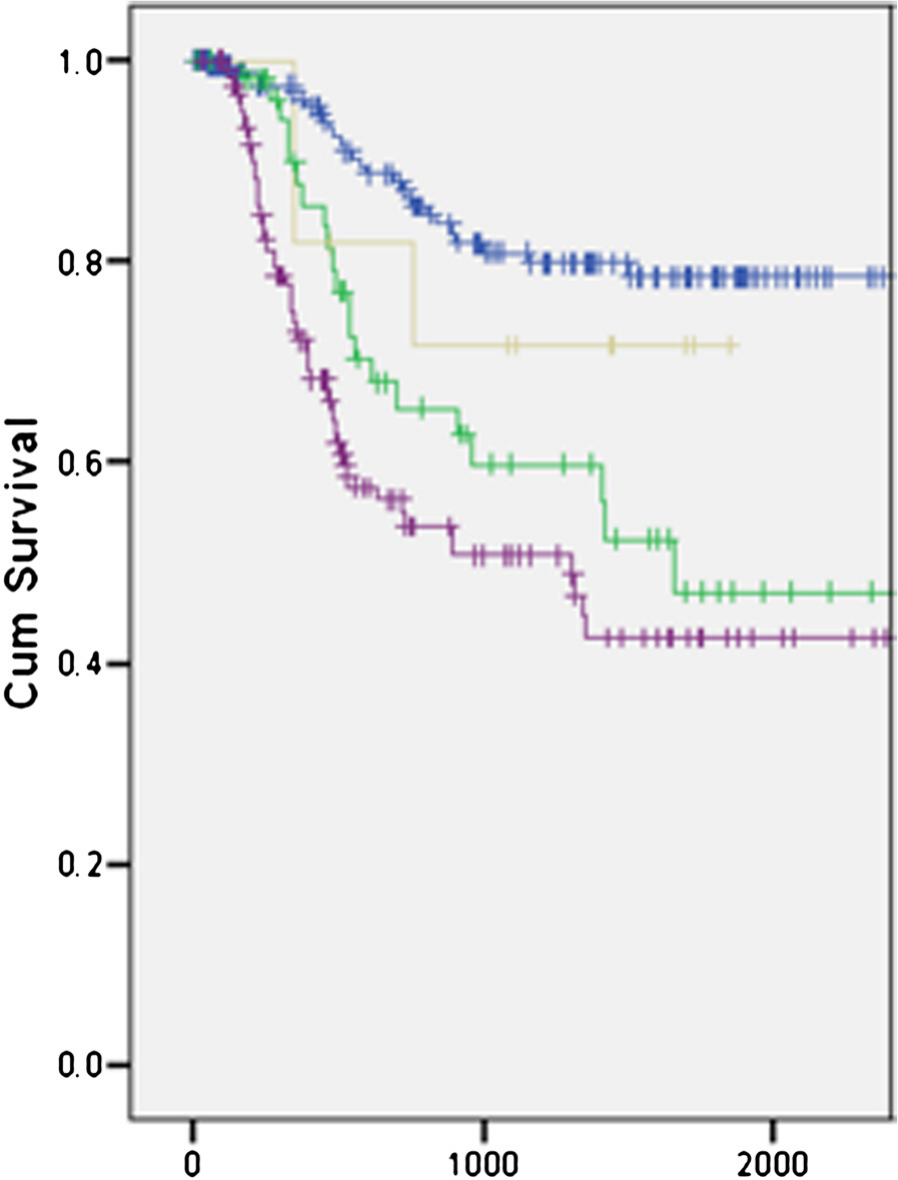
**Kaplan-Meir estimates of disease-specific survival according to stage discrepancy.** Blue, early stage patients with no change. Green, early stage patients upstaged to advanced stage. Purple, advanced stage patients with no change. Gold, advanced stage patients downstaged to early stage.

Given the significant differences in treatment between early and advanced stage patients, we compared survival between these groups as a function of staging discrepancy. Kaplan-Meir estimates of disease specific survival according to stage discrepancy is shown in Figure 2. In comparing the four groups described in Figure 1, a statistically significant difference in survival (p < 0.001) is present between these groups according to the Log-Rank test. However, there is no significant difference between early stage patients not upstaged and early stage patients that are upstaged. Similarly, no significant survival differences are shown between advanced stage patients that remained advanced stage following pathological analysis and downstaged patients. Cox-regression analysis incorporating age and gender also show no significant survival differences as a result of stage discrepancy.

## Discussion

Analyses of clinical and pathological correlations in oral carcinoma, such as positive margins, nodal status, extracapsular spread, degree of invasion and overall staging congruence are important in order to implement the most appropriate treatment pathways [5-7]. In our multi-institutional analysis of patients in Alberta, clinical and pathological staging was congruent in 21.9% of early stage patients upstaged and 7.9% of patients downstaged. Previous studies have shown the level of pathological upstaging in HNSCC patients with clinical N0 necks to be 34–44% [2,3], and an estimated 20–30% of OCSCC harbour occult regional metastases [8]. This is clinically relevant in the context of recommendations by the American Joint Committee on Cancer, which state an elective neck dissection (END) may be performed where the risk of nodal metastasis is greater than 20% [9]. A recent study also demonstrates that nodal disease is a strong independent predictor of outcome in OCSCC [10]. Taken together, although the level of upstaging in our study was relatively low, this lends support to perform END for OCSCC patients with clinically N0 necks.

Possible causes for staging discrepancy includes delay between clinical diagnosis and pathologic analysis resulting in upstaging, pathologic interpretation of specimen and lack of accuracy of clinical staging tools. Physical examination measures such as measurement of tumor and node size and manual palpation are relatively inaccurate and may be subjectively different based on surgeon experience. The lower limit of node palpation has been shown to be 0.5 cm in superficial areas and 1 cm in deeper regions [3]. The use of CT scanning does significantly improve the accuracy of staging, however, it does not detect micrometastasis and may have limited utility in differentiating nodal disease from submandibular gland in the submandibular region [3,11]. Therefore microscopic deposits and extracapsular spread may not be clinically identified and can only be definitively assessed by neck dissection with pathological assessment. Given the current limitation in clinical staging even in combination with advanced imaging technology, initial surgical intervention for all patients with OCSSC may be warranted [12]. Some patients with early stage disease only treated with radiation will not have the benefit of appropriate staging to initiate multimodality treatments known to improve survival in advanced stage OCSCC [13].

To our knowledge, the influence of clinical and pathological staging disparity on survival in OCSSC has not been reported. Our data suggests OCSSC patients pathologically upstaged or downstaged do not have a significantly altered disease-specific survival (Figure 2). It is important to note that all patients in this study had surgery as part of their treatment pathway, which is necessary to enable appropriate staging. In the 21.9% of patients with early stage disease, upstaging may have enabled for appropriate adjuvant treatment. In 7.9% of advanced stage patients, downstaging may have prevented unnecessary adjuvant treatment if initially treated surgically. Taken together, further study to determine the role of stage discrepancy on the alteration of treatment pathways way be warranted.

In contrast to other studies, although our data demonstrates staging discrepancy, we have found that this level of discrepancy does not significantly alter survival. One possibility for this result is a lower level of staging discrepancy in our cohort in comparison to other reports. In addition, most staging differences resulted in upstaging from early to advanced stage disease. In these cases, patients should have received appropriate post-operative radiation or chemoradiation and would therefore not be undertreated.

This study demonstrates levels of stage discrepancy in a cohort of patients predominantly treated with surgery as the primary treatment modality for OCSCC. This is in contrast with numerous practices in other institutions where chemoradiation is a first line treatment for OCSCC. In a subset of patients, primary surgical excision may provide more appropriate treatment if pathological upstaging or downstaging occurs from further analysis of the pathological specimen. For instance, when a patient is upstaged from early stage disease following surgery, chemoradiation may be added to the treatment protocol. Conversely, a patient being downstaged following surgery may have their therapy de-escalated. To further address these possibilities, a prospective analysis of patient outcomes following upstaging or downstaging should be performed.

Our study has a number of limitations. This is a retrospective analysis of patients staged by a variety of head and neck surgeons in various tertiary care centers, with specimen interpreted by different pathologists. This heterogeneity however enables a more realistic representation of overall staging differences. In terms of our survival analysis, one of the subgroups analysed, namely downstaged patients, was relatively small. This may therefore under represent a potentially significant difference in a larger sample size.

## Conclusions

Some disparity exists in clinical versus pathological staging in OCSCC, however, this does not have any significant impact on disease specific survival.

## Competing interests

The authors declare that they have no competing interests.

## Authors’ contributions

VB and DO performed data collection and statistical analysis. HS participated in data analysis. All authors contributed to drafting the manuscript. All authors read and approved the final manuscript.
